# The diabetes-associated K^+^ channel TALK-2 controls human beta cell endoplasmic reticulum Ca^2+^ handling, which promotes basal insulin release and limits glucose-stimulated insulin secretion

**DOI:** 10.1007/s00125-026-06683-9

**Published:** 2026-02-25

**Authors:** Jordyn R. Dobson, Prasanna K. Dadi, Matthew T. Dickerson, Arya Y. Nakhe, Soma Behera, Shannon E. Gibson, Spencer J. Peachee, Anthony Piron, Miriam Cnop, David A. Jacobson

**Affiliations:** 1 Department of Molecular Physiology and Biophysics, Vanderbilt University, Nashville, TN USA; 2 ULB Center for Diabetes Research, Université Libre de Bruxelles, Brussels, Belgium; 3 Interuniversity Institute of Bioinformatics in Brussels, Brussels, Belgium; 4 Pharmacognosy, Bioanalysis and Drug Discovery, Pharmacy Faculty, Université Libre de Bruxelles, Brussels, Belgium; 5 Division of Endocrinology, ULB Erasmus Hospital, Brussels University Hospital, Université Libre de Bruxelles, Brussels, Belgium; 6 WEL Research Institute, Wavre, Belgium

**Keywords:** Beta cell, Ca^2+^, ER, Insulin secretion, Ion channels, Islet, K2P, Live cell imaging, Single nucleotide polymorphisms, Type 2 diabetes

## Abstract

**Aims/hypothesis:**

The two-pore domain K^+^ channel TWIK1-related alkalinisation-activated K^+^ channel 2 (TALK-2) is encoded by *KCNK17*, which is one of the most abundant beta cell K^+^ channel transcripts that also shows high islet expression specificity. Polymorphisms that increase islet *KNCK17* expression or result in TALK-2 gain-of-function are associated with a predisposition for developing type 2 diabetes. However, there is a gap in knowledge of the beta cell function(s) of TALK-2. As K^+^ channels typically control beta cell Ca^2+^ handling, we aimed to examine the TALK-2 channel control of beta cell Ca^2+^ homeostasis and the resulting impact on insulin secretion.

**Methods:**

Localisation of TALK-2 was evaluated with immunofluorescent staining as well as TALK-2-GFP construct co-expressed with intracellular markers. TALK-2 function was evaluated by measuring changes in cytoplasmic Ca^2+^ (Ca^2+^_C_), endoplasmic reticulum Ca^2+^ (Ca^2+^_ER_), ER membrane potential (*V*_m_), K^+^ currents and insulin secretion in a TALK-2 inducible cell line and/or primary human beta cells with adenoviral-mediated shRNA knockdown (KD) of TALK-2 or scramble shRNA.

**Results:**

TALK-2 protein localised to the plasma membrane and ER membrane, and formed functional channels on the ER membrane. Ca^2+^_ER_ release was accelerated by TALK-2 (slope for TALK-2-expressing cells vs controls: 14.8 ± 0.7 vs 8.9 ± 1.3, respectively, shown as mean ± SE), which reduced Ca^2+^_ER_ storage (ΔCa^2+^_ER_ amplitude: TALK-2-expressing cells reduced by 25 ± 5%) and increased basal relative Ca^2+^_C_ (fold increase by 12 ± 2%). Furthermore, TALK-2 diminished ER membrane hyperpolarisation following Ca^2+^_ER_ release (Accelerated Sensor of Action Potentials [ASAP3_ER_] amplitude decreased by 20 ± 0.8% in TALK-2-expressing cells), suggesting that TALK-2 strengthens the electrical driving force for Ca^2+^_ER_ leak. In human beta cells, TALK-2-KD increased Ca^2+^_ER_ stores by reducing Ca^2+^_ER_ leak (2.30 ± 0.12 vs controls 2.65 ± 0.14). Moreover, TALK-2-KD reduced beta cell Ca^2+^_C_ at euglycaemic conditions (2.88 ± 0.36 vs controls 3.16 ± 0.36) and increased beta cell Ca^2+^_C_ influx in response to hyperglycaemic conditions (4.07 ± 0.55 vs controls 3.45 ± 0.48). Human pseudoislets with beta cell-specific TALK-2-KD displayed reduced basal insulin secretion (0.266 ± 0.065 vs controls 0.432 ± 0.073) and enhanced glucose-stimulated insulin secretion (GSIS; 85.01 ± 13.96 vs controls 42.53 ± 5.52).

**Conclusions/interpretation:**

These data support the notion that TALK-2 functions on the human beta cell ER membrane to increase the electrical driving force for beta cell Ca^2+^_ER_ release, reduces glucose-stimulated Ca^2+^ influx and limits GSIS. Furthermore, TALK-2-mediated amplification of Ca^2+^_ER_ leak likely enhances basal insulin secretion by increasing Ca^2+^_C_. Therefore, polymorphisms in *KCNK17* that increase TALK-2 activity or expression would be predicted to increase type 2 diabetes risk by blunting beta cell glucose-stimulated Ca^2+^ influx, limiting GSIS, promoting Ca^2+^_ER_ leak and elevating basal insulin secretion.

**Graphical Abstract:**

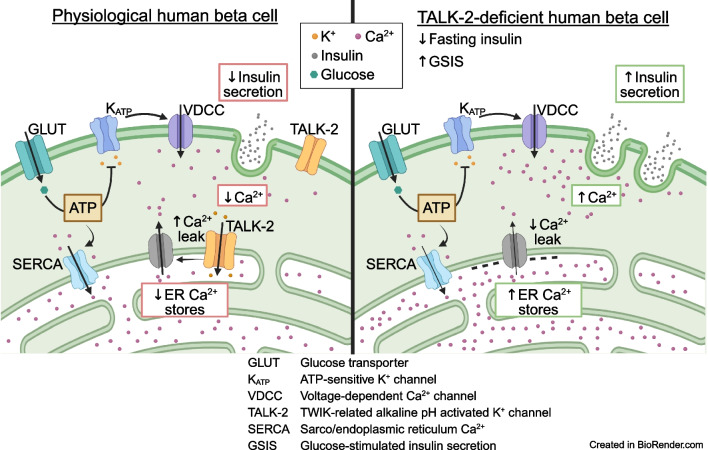

**Supplementary Information:**

The online version contains peer reviewed but unedited supplementary material available at 10.1007/s00125-026-06683-9.


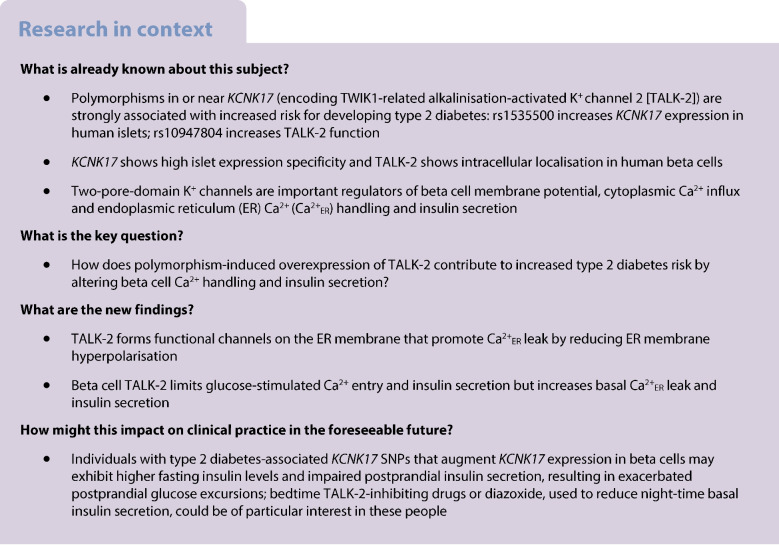


## Introduction

Endoplasmic reticulum (ER) Ca^2+^ (Ca^2+^_ER_) serves critical roles in beta cell function such as cytoplasmic and mitochondrial Ca^2+^ handling, protein folding and the unfolded protein response (UPR) [1–4]. Ca^2+^_ER_ is controlled by ion channels both at the plasma membrane and the ER membrane. Beta cell ER and cytoplasmic Ca^2+^ (Ca^2+^_C_) handling are disrupted during the pathogenesis of diabetes and thus SNPs in or around the genes encoding several ion channels have been associated with increased type 2 diabetes risk [5, 6]. Some of these genes include those encoding K^+^ channels (*KCNJ11*, *KCNQ1*, *KCNK16* and *KCNK17*) and Ca^2+^ channels (*CACNA1D*, *ATP2A1*, *ATP2A3* and *ITPR2*), many of which have roles controlling Ca^2+^_ER_ handling or storage. However, the beta cell function(s) of *KCNK17* remain elusive.

There are at least nine type 2 diabetes-associated SNPs in the promoter and coding region of *KCNK17*, which encodes the TWIK1-related alkalinisation-activated K^+^ channel (TALK) 2 (rs1535500, rs10947804, rs12663159, rs146060240, rs3734618, rs11756091, rs11756070, rs11753141 and rs34247110) [7]. These polymorphisms have been shown to either increase islet *KCNK17* expression or result in TALK-2 gain-of-function (GOF) [7, 8]. TALK-2 channels are also activated by diabetogenic conditions such as elevated long-chain fatty acid coenzyme As and reactive oxygen species (ROS) [9–11]. TALK-2 activation during beta cell fatty acid metabolism and ROS generation may reduce beta cell function to a greater degree in individuals with type 2 diabetes-associated *KCNK17* SNPs. However, studies on TALK-2 in beta cells have been hindered due to a lack of selective pharmacology and absence of the *KCNK17* gene in the rodent genome [12].

The two-pore domain K^+^ (K2P) channels TALK-1 and TALK-2 show the most islet-restricted expression of all ion channels associated with diabetes [5, 7]. Additionally, the RNA levels of the genes encoding TALK-1 and TALK-2 are the first and third most abundant K^+^ channel transcripts in human beta cells, respectively [13]. TALK-1 and TALK-2 channels are located adjacent to each other on chromosome 6 and show strong sequence homology (60%) [14]. Although the function of beta cell TALK-2 has not been determined, TALK-1 channels on the plasma membrane are important for hyperpolarising the plateau potential from where action potentials fire, which limits Ca^2+^ influx and insulin secretion [15]. Because TALK-2 channels become active at supraphysiological alkaline pH levels, plasma membrane TALK-2 homodimers may not be functional under physiological pH conditions [16]. However, TALK-1 and TALK-2 α-subunits can form heterodimeric channels that are active at physiological pH but show reduced conductance compared with TALK-2 homodimers [17]. Interestingly, TALK-1/TALK-2 heterodimers and TALK-2 homodimers show intracellular localisation in the human beta cell line EndoC-βH1 and following heterologous expression, respectively [17, 18]. As ER-localised TALK-1 channels control Ca^2+^_ER_, intracellular TALK-2 may also regulate intracellular Ca^2+^ stores [18, 19].

While the importance of precise beta cell Ca^2+^_ER_ handling has been well established, most of the literature has focused on Ca^2+^ pumps and Ca^2+^ channels [4, 20–24]. However, it has become increasingly clear that K^+^ channels play an important role in maintaining the electrical driving force for Ca^2+^_ER_ release. These ER-localised K^+^ channels provide a K^+^ countercurrent that dissipates the buildup of negative charge on the ER membrane during Ca^2+^ release [5, 25, 26]. Some of these channels include trimeric intracellular cation (TRIC)-A and -B channels, small-conductance Ca^2+^-activated K^+^ (SK_Ca_) channels, large-conductance Ca^2+^-activated K^+^ (BK_Ca_) channels and K2P channels (e.g. TASK-1 and TALK-1) [18, 25, 27, 28]. For example, TALK-1 forms functional channels on the beta cell ER membrane; flux through these channels limits Ca^2+^_ER_ storage by promoting Ca^2+^_ER_ leak, which elevates Ca^2+^_C_ and sensitises beta cells to ER stress [18]. Importantly, a diabetes-associated non-synonymous SNP in *KCNK16*, resulting in TALK-1 GOF, diminished beta cell Ca^2+^_ER_ storage and promoted ER stress under diabetogenic conditions [18, 29, 30]. Thus, it is important to determine whether intracellular TALK-2 or TALK-1/TALK-2 channels serve a role in regulating beta cell Ca^2+^_ER_.

## Methods

### Co-localisation analysis

We applied the *colocRedRibbon* v1.1 R package to identify shared causal variants between multi-ancestry type 2 diabetes genome-wide association studies (GWAS) and human islet expression quantitative trait locus (eQTL) summary statistics [31–33]. Variants were first aligned by effect allele and split based on the direction of eQTL effect relative to the GWAS risk allele. Within each set, variants were ranked by *p* value, and the *RedRibbon* method was used to detect overlap of top-ranked variants via a hypergeometric test [34]. Overlapping variants form a candidate set for co-localisation analysis using the coloc package v5.2.3, which estimates the posterior probability of shared causality. Analyses were conducted in R using *colocRedRibbon* v1.1 [35].

### Chemicals and reagents

All chemicals and reagents were purchased from Thermo Fisher or Sigma-Aldrich unless specified otherwise.

### Human islets

Human islets were obtained from several isolation centres through the Integrated Islet Distribution Program (IIDP). Informed consent for deceased donors was obtained by the IIDP in accordance with the National Institute of Health. These studies were approved by the Vanderbilt University Health Sciences Committee Institutional Review Board (IRB no. 110164). Deidentified human donor information is provided in electronic supplementary material (ESM) Table 1. The human islets checklist can be found at the end of the ESM file. For immunofluorescent staining, deidentified human pancreas samples came from the National Cancer Institute-funded Cooperative Human Tissue Network (CHTN).

### Cell culture

The generation of stable T-REx-293 cell lines containing the pcDNA5/TO *KCNK17* plasmid (TALK-2) has been described previously [36]. T-REx-293 cells were cultured at 37°C with 5% CO_2_ in DMEM + GlutaMAX media (Gibco, catalogue no. 10569010) with 50 U/ml penicillin, 50 µg/ml streptomycin, 250 µg/ml hygromycin, 5 µg/ml blasticidin S and 10% FBS. TALK-2 expression was induced with tetracycline (1 µg/ml) 18–24 h prior to experiments. INS-1 (832/13) cells were cultured at 37°C with 5% CO_2_ in RPMI media (Gibco, catalogue no. 11875093) supplemented with 10 mmol/l HEPES, 1 mmol/l sodium pyruvate, 50 μmol/l β-2-mercaptoethanol and 10% FBS.

### Human pseudoislet formation

Human pseudoislets were formed as described previously [37]. Human islets were picked into 1.5 ml centrifuge tubes and spun at 300 *g* for 3 min. Islets were washed with Versene (Gibco, catalogue no. 15040066) and dispersed using prewarmed trypLE (Gibco, catalogue no. 12604-13). PBS containing 5% FBS (MACS buffer) was used to neutralise trypLE and for magnetic sorting. Dispersed islet cells were then incubated with monoclonal mouse NTPDase3 antibody (5μg/ml; RRID:AB_2752250; Quebec, Canada) with rotation at 4°C for 30 min. After washing with MACS buffer, islet cells were incubated with anti-mouse IgG2a+b MicroBeads (Miltenyi Biotec, catalogue no. 130-047-202) for 15 min at 4°C. Islet cells were washed with MACS buffer and positive sorting of beta cells was done using the QuardoMACS separator (Miltenyi Biotec). Beta cells were transduced with either shScramble or sh*KCNK17* adenovirus (containing mKate2 fluorescent reporter; transduced at 100 multiplicity of infection [MOI]; VectorBuilder) (targeting sequences: shScramble ACCTAAGGTTAAGTCGCCCTCG; sh*KCNK17* GGATGTCGTCCAAGCATACAAA, CCGCCTCTTCTGCATCTTCTTT and GCGACTACGTGATTGGAATGAA) in a non-tissue culture-treated 24-well plate for 2 h. Beta cells were collected and washed with CMRL media (Gibco, catalogue no. 11530–037) containing 20% FBS, 100 μg/ml penicillin/streptomycin, 2 mmol/l GlutaMAX, 2 mmol/l HEPES and 1 mmol/l sodium pyruvate. Beta cells were combined with the non-beta cell fraction (60% beta cells and 40% non-beta cells) and plated in a 96-well plate containing microwells (EZSHERE SP Microplate; IWAKE=, catalogue no. 4860-900sp). Media was exchanged every 2 days. Pseudoislets were formed and used for experiments 6–7 days after plating.

### Immunofluorescence

Paraffin-embedded human pancreas sections were rehydrated by submerging in xylene, 100% EtOH, 90% EtOH, 70% EtOH, 50% EtOH and H_2_O, respectively. For antigen retrieval, the sections were placed in boiling citric acid-based antigen unmasking solution (Vector Laboratories, catalogue no. H-3300) buffer for 6 min. Sections were then blocked in 2% normal donkey serum (NDS; Jackson ImmunoResearch Laboratories), 1% BSA in PBS-T for 1 h at room temperature. Primary antibodies were added and incubated overnight at 4°C: 1:1000 guinea pig anti-insulin (catalogue no. 20-IP35; Fitzgerald); 1:200 mouse anti-TALK-2 (catalogue no. sc-390435, Santa Cruz); and 1:200 rabbit anti-GRP94 (catalogue no. NB300-619, Novus Biologicals). Sections were washed and secondary antibodies (1:300 donkey anti-mouse Alexa Fluor 647 [catalogue no. 715-606-150; Jackson ImmunoResearch], donkey anti-guinea pig Alexa Fluor 488 [catalogue no. 706-546-148; Jackson ImmunoResearch], and donkey anti-rabbit Alexa Fluor Cy3 [catalogue no. 711-166-152]) were incubated in the dark at room temperature for 2 h. Images were obtained using a Zeiss LSM 710 META inverted confocal microscope.

### Real-time quantitative PCR

RNA was isolated from dispersed human islets that were transduced with either shScramble or sh*KCNK17* adenovirus. cDNA was generated following manufacturer’s protocol (iScript cDNA synthesis kit, catalogue no. 1708890, BioRad). Real-time quantitative PCR (qPCR) was performed using 100 ng of cDNA and iTaq Universal SYBR Green supermix (catalogue no. 1725120, BioRad) and primers for *KCNK17* (forward 5′ GAGCTGTTGCAGAACTTCAC 3′; reverse 5′ GGTGATGGTGGACACAGAAA 3′) and GAPDH (forward 5′ AGCCACATCGCTCAGACAC 3′; reverse 5′ GCCCAATACGACCAAATCC 3′).

### Live cell imaging

#### Thallium (Tl^+^) flux assay

The Tl^+^ flux assay was performed as described previously [36]. Briefly, TALK-2 inducible cells were plated on a 384 well plate (Corning, catalogue no. 356719) at 25,000 cells/well. Tetracycline (1 μg/ml) was added to half of the wells to induce TALK-2 expression 24 h prior to imaging. On the day of imaging, cells were loaded with 2 μmol/l Thallos dye in assay buffer (1× HBSS containing 20 mmol/l HEPES, pH 8.8) and 0.01% Pluronic F-127 for 1 h at 37°C and 5% CO_2_. Cells were washed in assay buffer and imaged with a whole-plate kinetic imager (Panoptic, Wavefront Bio). The Tl^+^ stimulus buffer (5× in mmol/l: 125 d-gluconic acid Na^+^ salt, 1 MgSO_4_, 1.8 CaSO_4_, 5 d-(+)-glucose, 20 HEPES, 7.5 Tl_2_SO_4_) was added to the wells after 10 s of baseline imaging. Thallos fluorescence was monitored with excitation λ 482 nm and emission λ 536 nm.

#### Cytoplasmic Ca^2+^, Ca^2+^_ER_ and ER *V*_m_ imaging

Cells were loaded for 20 min with Fura2-AM (2 μmol/l) Ca^2+^_C_ dye at 37°C and 5% CO_2_. Fura2-AM fluorescence was measured ratiometrically at excitation λ 340 nm and 380 nm, and emission λ at 535 nm. Confocal microscopy was used for imaging genetically encoded Ca^2+^ indicators (ER-localised GFP-Ca^2+^-measuring organelle-Entrapped Protein IndicAtor 1 fused with a SNAP tag [GCEPIA1-SNAP_ER_] for Ca^2+^_ER_, Green Calcium Modulated Protein, version 6, slow [GCaMP6s] for Ca^2+^_C_) and ER-specific genetically encoded voltage indicator (Accelerated Sensor of Action Potentials [ASAP3_ER_]) [18, 38–41]. Cells were transduced with GCaMP6s (excitation λ 488 nm, emission λ 531 nm) virus for 4 h and were imaged 2 days post-transduction. ASAP3_ER_ (excitation λ 488 nm, emission λ 531 nm) and GCEPIA1-SNAP_ER_ (GCEPIA1: excitation λ 488 nm, emission λ 531 nm) were transfected into cells 2 days before imaging using Lipofectamine3000 as per manufacturer’s protocol. For experiments using GCEPIA1-SNAP_ER_, SNAP-Cell 647-SiR substrate (3 μmol/l; SNAP tag: excitation λ 645 nm, emission λ 661 nm) was loaded into cells for 30 min. Cells were washed and incubated for 10–20 min (experiment dependent) in KRB containing (in mmol/l) 119 NaCl, 10 HEPES, 4.7 KCl, 2 CaCl_2_, 1.2 MgSO_4_, and 1.2 KH_2_PO_4_ (pH 7.35 adjusted by NaOH). CaCl_2_ was excluded from the extracellular buffer for experiments assessing Ca^2+^_ER_ and changes in ER membrane voltage. During each experiment, cells were treated as defined in the figures. Images were taken every 5 or 10 s with a Nikon Ti2 epifluorescence microscope equipped with Photometrics Prime 95B 25 mm sCMOS Camera (for Fura2-AM), LSM 780 multi-photon confocal microscope (for GCamP6S) or Nikon Crest V3 spinning disk confocal with a Ti2-E microscope (for GCEPIA1-SNAP_ER_ and ASAP3_ER_).

### Dynamic insulin secretion

For insulin secretion assays, human pseudoislets were equilibrated in DMEM supplemented with 0.5 mg/ml BSA, 0.5 mmol/l CaCl_2_ and 10 mmol/l HEPES for 2 h at 37°C and 5% CO_2_. Sixty pseudoislets were loaded per chamber and immobilised with Bio-Gel P-4 Media (catalogue no. 150-4124, BioRad). Pseudoislets were perifused at a flow rate of 100 μl/min in the indicated solutions at 37°C with the perifusate collected at 4°C (PERI-Lite perifusion system, BioRep). Insulin concentrations were determined with ELISA (catalogue no.10-1113-01, Mercodia). Total pseudoislet insulin was extracted in acid ethanol (1.5% HCl in 95% EtOH) at 4°C for 24 h and determined with ELISA.

### Electrophysiology

For electrophysiology recordings, an Axopatch 200B amplifier with pCLAMP10 software (v. 10.7; Molecular Devices) was used. Single sorted beta cells were washed and recorded in Krebs Ringer buffer (pH 7.4) with 11 mmol/l glucose and the K^+^ channel inhibitors: tetraethylammonium chloride hydrate (TEA) (10 mmol/l) and tolbutamide (100 μmol/l). Patch electrodes were pulled to a resistance of 3–5 MΩ and loaded with intracellular solution containing (in mmol/l) 140 KCl, 1 MgCl_2_, 10 EGTA, 10 HEPES and 4 MgATP (pH adjusted with KOH to 7.2). The osmolarity was kept the same between the extracellular and intracellular buffers (305–315 Osm/l). The whole-cell voltage clamp configuration was used to record K2P currents with a voltage ramp from −120 mV to 60 mV every 15 s [30]. Currents were digitised using Digidata 1440, low-pass-filtered at 1 kHz and sampled at 10 kHz.

Single-channel recordings of ER-localised TALK-2 channels were accomplished using the nuclear patch-clamp technique as described previously [18, 42, 43]. Cells were split with trypsin and neutralised with media. These cells were then resuspended in nuclear isolation solution containing KCl, sucrose, Tris-HCl, β-mercaptoethanol and phenylmethylsulfonyl fluoride. A dounce homogeniser was used to disrupt the plasma membrane to release nuclei into the solution. The nuclei were plated on an imaging dish containing intracellular solution (in mmol/l: 150 KCl, 10 HEPES, 0.5 EGTA and 0.36 CaCl_2_). Patch pipettes were pulled to a resistance of 8–10 MΩ, loaded with intracellular solution and 4 mmol/l NaATP, and coated with Sigmacote. Single-channel recordings were low-pass-filtered at 1 kHz and sampled step-wise (increments of 25 mV) at 50 kHz from 100 mV to −100 mV for 10 s each.

### Statistical analysis

Analysis was conducted using GraphPad Prism (v.10.6.1), Excel (v. 2511) or Clampfit (v. 11.2). Data are presented as mean ± SE. Statistical significance was determined by conducting two-tailed *t* tests (unpaired or paired as specified), one-way ANOVA, one-tailed *t* test or multiple *t* tests. Each data point on a bar graph represents a biological replicate; these points are the mean of two or three technical replicates and are indicated in the results and figure legends. For human islet experiments, sex was determined by clinical examination, and this information is included in ESM Table 1. Data for islets from both sexes were all included in data analysis. There was no exclusion of islet cohorts from analysis post-experimentation.

## Results

### rs34247110 is a type 2 diabetes risk SNP through modulation of human islet *KCNK17* expression

**Fig. 1:**
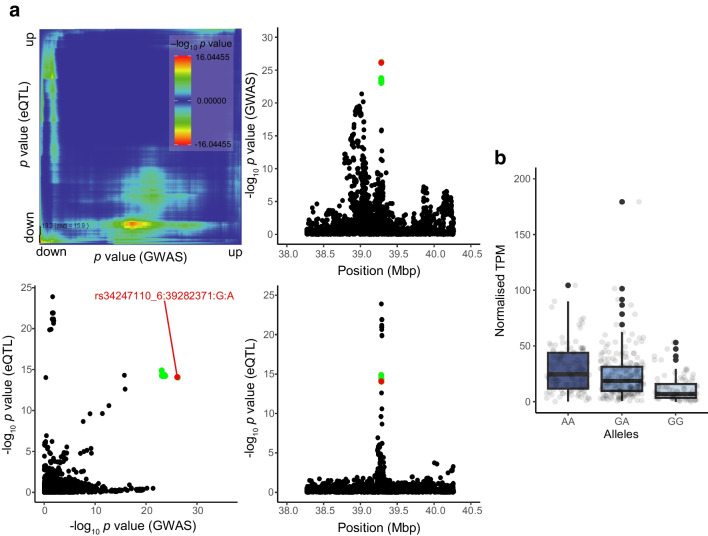
rs34247110 is a type 2 diabetes risk SNP candidate through upregulation of *KCNK17*. (**a**) The overlap of GWAS and eQTL signals, highlighting the most significant shared SNPs (the coloured circles on subsequent panels). Analysis was performed within 2 million base pairs centred on the chromosomal region of *KCNK17* transcription start site. The lead variant, rs34247110 (red circle), has risk allele A, which increases both type 2 diabetes risk and *KCNK17* expression (posterior probability 0.45). The 99% credible set is shown in green. (**b**) *KCNK17* expression (in transcripts per millions, TPM) with A or G alleles of rs34247110 identified by *colocRedRibbon*. The horizontal bar represents the median. The first (Q1) and third (Q3) quartiles are identified by the lower and upper hinges, respectively. Whiskers extend to the most extreme data points within 1.5 × IQR below Q1 and above Q3. The black dots indicate potential outliers, and translucent grey dots represent individual observations for 404 eQTL samples

GWAS have identified SNPs in and around the *KCNK17* gene as being associated with type 2 diabetes risk. To examine whether these SNPs exert their effects through modulation of *KCNK17* expression levels, we performed a co-localisation analysis of multi-ancestry GWAS and human islet eQTL SNPs [31, 32] using the *colocRedRibbon* tool [33]. The *colocRedRibbon* framework delineates overlapping association signals across a 2 million bp region flanking *KCNK17* by ranking *p* values from eQTL and GWAS analyses and using the *RedRibbon* method [34] to find common signals (Fig. 1a). The 99% credible set (green circles, Fig. 1a) refines the shared causal variants, and the red circle in the figure represents the lead variant, rs34247110. *colocRedRibbon* demonstrated strong evidence for shared causal mechanisms, with a co-localisation posterior probability (PP.H4.abf) of 0.99, indicating a high likelihood that the same genetic variant influences both diabetes risk and *KCNK17* expression; the lead variant rs34247110 showed a posterior probability (SNP.PP.H4) of 0.45 within this shared signal. rs34247110 maps to an active promoter region, consistent with its regulatory role in *KCNK17* expression. The analysis revealed that the diabetes risk allele A of variant rs34247110 is associated with increased *KCNK17* gene expression in human islets (Fig. 1b). These findings are consistent with and extend the results reported by Varshney et al who performed systematic co-localisation of islet eQTLs with type 2 diabetes GWAS signals and identified several loci with shared genetic regulation [7]. While Varshney et al highlighted four SNPs having a potential impact on gene expression, our *colocRedRibbon* approach provided higher-resolution mapping of the shared signal at the *KCNK17* locus, pinpointing rs34247110 as a likely causal variant with strong statistical support.

### TALK-2 is localised and functional on the ER and plasma membrane

**Fig. 2:**
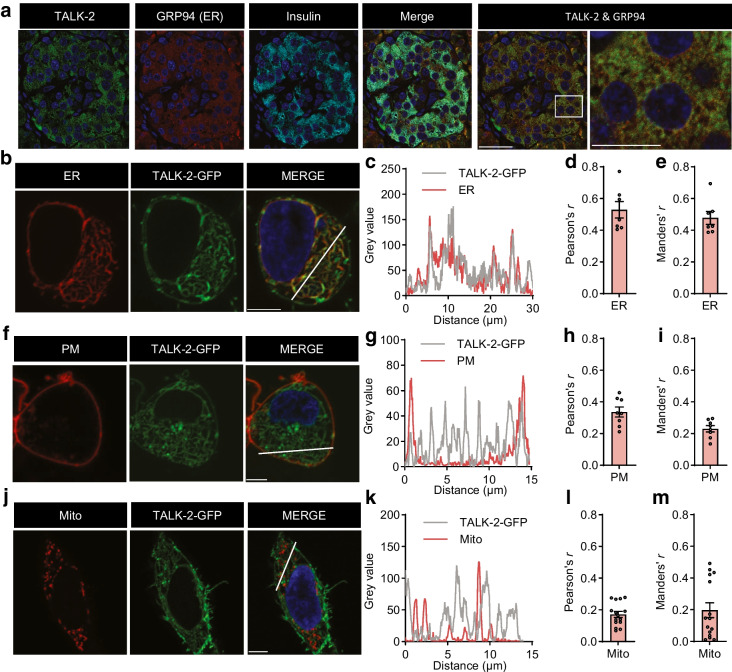
TALK-2 channels are localised to the plasma membrane and the ER membrane. (**a**) Immunofluorescent staining of a human pancreas section probed for TALK-2, GRP94 and insulin. (**b**–**e**) Live HEK cells transfected with TALK-2-GFP and ER-RFP were subjected to immunofluorescent staining (**b**). Linescans of fluorescence intensity profiles between the red and green channels indicated by the white lines in (**b**) are shown (**c**), as well as Pearson’s *r* (**d**) and Manders’ *r* (**e**) between TALK-2-GFP and ER-RFP (*n*=7 cells). (**f**–**i**) Live HEK cells transfected with TALK-2 and plasma membrane dye were subjected to immunofluorescent staining (**f**). Linescans of fluorescence intensity profiles between the red and green channels in (**f**) are shown (**g**), as well as Pearson’s *r* (**h**) and Manders’ *r* (**i**) between TALK-2-GFP and plasma membrane dye (*n*=8 cells). (**j**–**m**) Live HEK cells transfected with TALK-2-GFP and Mito-mOrange2 were subjected to immunofluorescent staining (**j**). Linescans of fluorescence intensity profiles between the red and green channels in (**j**) are shown (**k**), as well as Pearson’s *r* (**l**) and Manders’ *r* (**m**) between TALK-2-GFP and Mito-mOrange2 (*n*=15 cells). Scale bar, 30 μm (**a**) or 5 μm (**b**, **f**, **j**). The white box indicates the area observed in the magnified image on the right. Scale bar of rightmost image is 13 μm. Data are presented as mean with SE. Mito, Mito-mOrange2; PM, plasma membrane

Human pancreatic sections stained for TALK-2 showed intracellular staining in insulin-positive beta cells that co-localised with an ER-resident protein, glucose-regulated protein 94 (GRP94) (Fig. 2a). Heterologous expression of a TALK-2-GFP construct showed strong co-localisation coefficients with ER-RFP (Fig. 2b–e), intermediate co-localisation with a plasma membrane dye (CellBrite Steady 550; Fig. 2f–i) and low co-localisation with Mito-mOrange2 and nuclei (Fig. 2j–m). This suggests that TALK-2 primarily localises to the ER and partially to the plasma membrane.

**Fig. 3:**
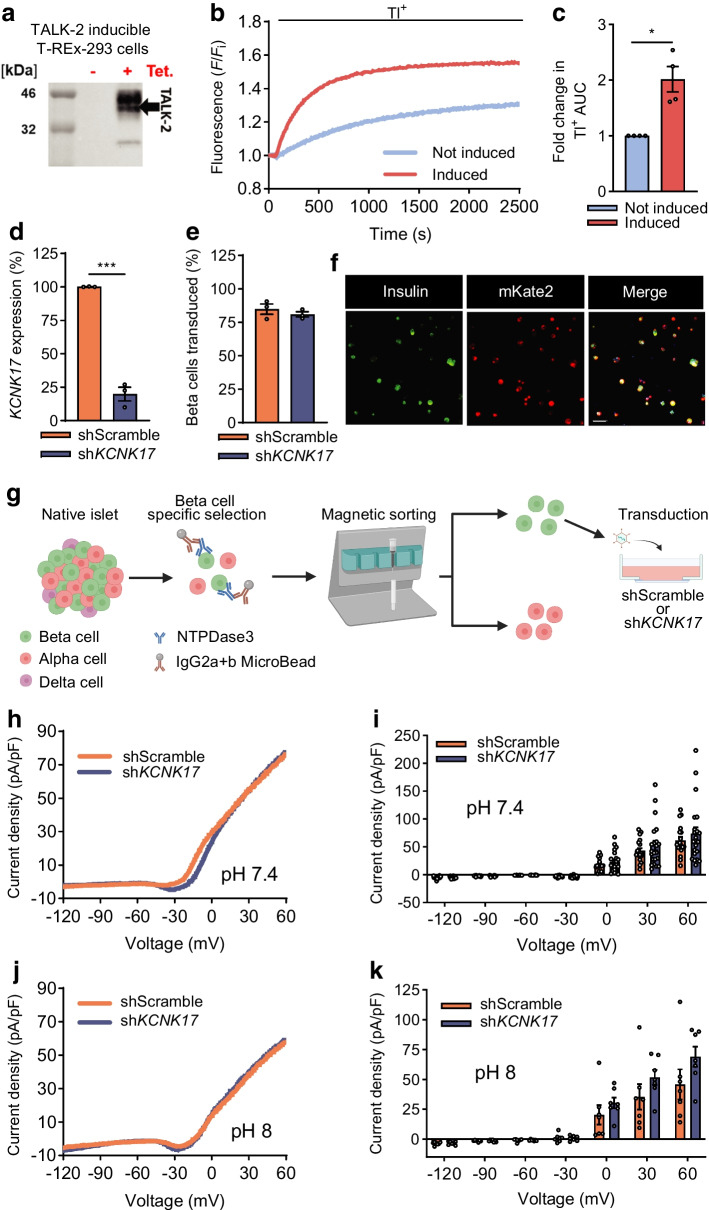
TALK-2 channel activity on the plasma membrane of inducible cells and human beta cells. (**a**) Western blot of T-REx-293 cell lysate probed with TALK-2 antibody to confirm tetracycline-inducible system (left, ladder; middle, no induction; right, tetracycline induction). (**b**) Representative traces from a Tl^+^ flux assay of induced TALK-2 cells and not-induced cells at a pH of 8.8. Data were normalised to Thallos dye fluorescence at the initial timepoint (fluorescence/initial fluorescence [*F*/*F*_i_]). (**c**) Fold change in Tl^+^ AUC relative to the not-induced cells (*n*=4 biological replicates). (**d**) *KCNK17* expression from dispersed human islets transduced with either shScramble or sh*KCNK17* (*n*=3 donors). (**e**) Percentage of beta cells that were transduced with either shScramble or sh*KCNK17* (*n*=3 donors). (**f**) Immunofluorescent staining of dispersed islets stained for insulin and TagRFP (to detect the fluorescent reporter mKate2). Scale bar, 100 μm. (**g**) Schematic showing dispersion and magnetic sorting of human islets for electrophysiology of beta cells with and without TALK-2-KD (created in BioRender. Dobson, J. (2026) https://BioRender.com/cr41lz5). (**h**, **j**) Representative K2P current density traces obtained from dispersed beta cells transduced with either shScramble or sh*KCNK17* at pH 7.4 (**h**) and pH 8 (**j**). (**i**, **k**) The current density of K2P channels when the membrane potential was held at the specific voltages between shScramble or sh*KCNK17* beta cells at pH 7.4 (shScramble *n*=18 beta cells; sh*KCNK17 n*=22 beta cells) (**i**) and pH 8 (shScramble *n*=7 beta cells; sh*KCNK17 n*=7 beta cells) (**k**). Data points on bar graphs represent biological replicates (**c**), donors (**d**, **e**), or cells (**i**, **k**). *p<0.05, ***p<0.001 (by one-sample *t* test [**c**], unpaired *t* test [**d**, **e**] or multiple unpaired *t* tests [**i**, **k**]). Tet, tetracycline

TALK-2 channel activity was investigated using a tetracycline-regulated TALK-2 cell line, which exhibited strong tetracycline induction of TALK-2 expression (Fig. 3a). Plasma membrane TALK-2 activity was assessed with a thallium (Tl^+^) flux assay that revealed significant Tl^+^ influx with TALK-2 expression (induced) in comparison with controls (not induced) (twofold increase in Tl^+^ flux; *n*=4; Fig. 3b, c). Primary human beta cell TALK-2 function was examined using whole-cell patch-clamp to determine plasma membrane activity in cells expressing either Scramble or *KCNK17* specific shRNA(s) (knockdown of 80.16 ± 5.07% of *KCNK17* expression in sh*KCNK17* transduced islet cells; beta cell transduction efficiency of 84.88 ± 3.88% in shScramble and 80.92 ± 1.95% in sh*KCNK17*; *n*=3 Fig. 3d–f). Interestingly, there was no change in K2P currents in beta cells expressing sh*KCNK17* compared with shScramble (Fig. 3g–k). Although this suggests that TALK-2 is not functional on the beta cell plasma membrane, there could be compensation by other K2P channels and/or the recording solutions used did not contain conditions required for TALK-2 activity.

**Fig. 4:**
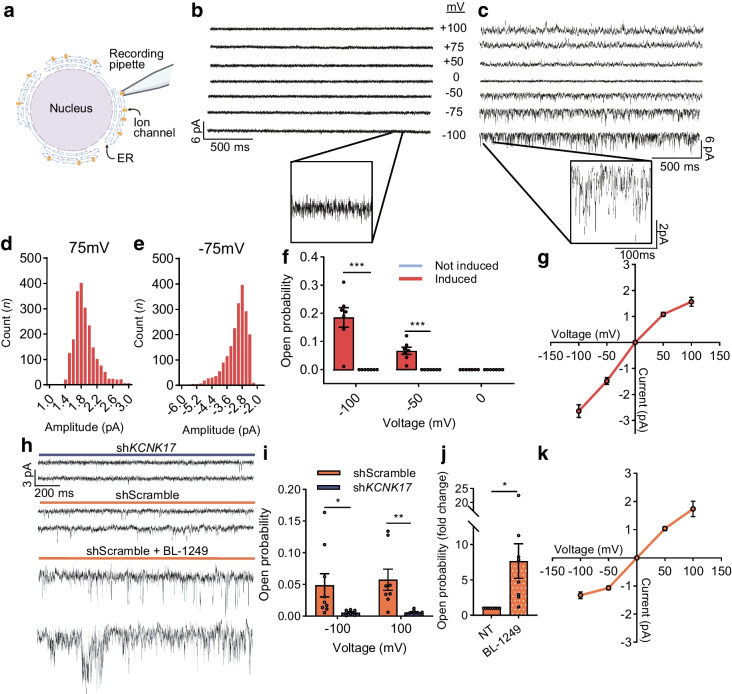
TALK-2 channels are functional on the ER membrane. (**a**) Schematic depicting nuclear patch-clamp technique for recording single-channel currents of ER-localised ion channels from T-REx-293 or human beta cells (created in BioRender. Dobson, J. (2026) https://BioRender.com/49y395l). (**b**, **c**) Representative single-channel current recordings from nuclei of a not-induced cell (**b**) and induced TALK-2 cell (**c**) at the specified voltages. (**d**, **e**) Histograms for the count (over 3 s duration) vs amplitude (pA) of the TALK-2 currents detected at 75 mV (**d**) and −75 mV (**e**) (*n*=5 nuclei). (**f**) Open probability of TALK-2 induced vs not-induced cells at the indicated voltages. (**g**) I–V curve of TALK-2-expressing cells (*n*=7 nuclei from both TALK-2-expressing and not-induced cells). (**h**) Representative single-channel recordings at −100 mV from nuclei isolated from beta cells transduced with either sh*KCNK17* or shScramble, as well as shScramble cells treated with BL-1249. (**i**) Open probability analysis of TALK-2-like currents from nuclei isolated from sh*KCNK17* or shScramble human beta cells at −100 mV (*n*=9 nuclei) and 100 mV (*n*=8 nuclei). (**j**) Fold increase in open probability from shScramble beta cells treated with BL-1249 (*n=*7 nuclei). (**k**) I–V curve of shScramble beta cells (*n*=5–9 nuclei). *p<0.05, **p<0.01, ***p<0.001 (by multiple unpaired *t* test [**f**, **i**] or one-sample *t* test)

Nuclear patch-clamp recordings also assessed TALK-2 function on the ER membrane surrounding the nucleus from both T-REx-293 cells and human beta cells (Fig. 4). K^+^ permeable single-channel currents resembling TALK-2 amplitude and open probability were present on ER recordings from cells expressing TALK-2 but were undetectable in ER membranes from control cells without TALK-2 expression (open probability at −100 mV induced 0.185 ± 0.035 vs not-induced 0.00 ± 0.00; at −50 mV induced 0.067 ± 0.013 vs not-induced 0.00 ± 0.00; Fig. 4b–g; *n*=7 nuclei/condition). K2P single-channel currents resembling TALK-2 were also found on the ER membrane from control human beta cells, which exhibited a reduction in nuclei from TALK-2-KD beta cells (open probability at −100 mV shScramble 0.0433 ± 0.0183 vs sh*KCNK17* 0.0053 ± 0.0013; at 100 mV shScramble 0.0575 ± 0.0144 vs sh*KCNK17* 0.0057 ± 0.0012; Fig. 4h, i; *n*≥8). Moreover, the single-channel currents resembling TALK-2 were significantly increased following the addition of the TALK-2 agonist BL-1249 (10 μmol/l; 7.66 ± 2.44-fold increase in open probability; Fig. 4h, j).

### TALK-2 increases Ca^2+^_ER_ release, elevates store-operated Ca^2+^ entry and diminishes Ca^2+^_ER_ stores by dissipating negative charge on the ER luminal membrane

**Fig. 5:**
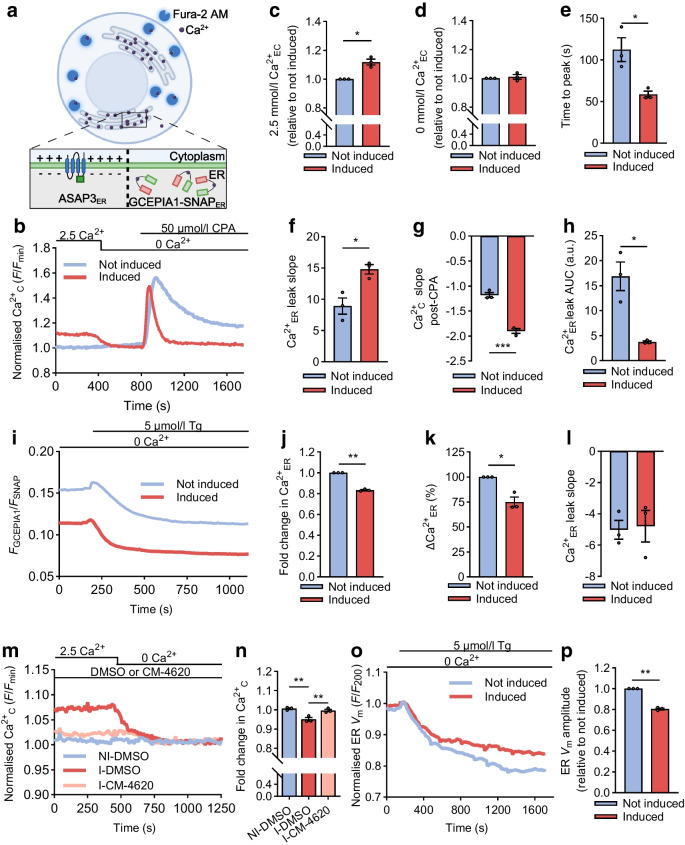
TALK-2 reduces Ca^2+^_ER_ storage, increases SOCE and limits hyperpolarisation of the ER membrane. (**a**) Schematic representing the indicators used to evaluate TALK-2 modulation of Ca^2+^_ER_ stores: Fura2-AM is a Ca^2+^_C_ indicator; GCEPIA1-SNAP_ER_ is a genetically encoded ER-localised ratiometric Ca^2+^ sensor; and ASAP3_ER_ is a genetically encoded ER-localised voltage indicator (created in BioRender. Dobson, J. (2026) https://BioRender.com/96e5ax7). (**b**) Representative Ca^2+^_C_ traces for TALK-2-expressing cells and not-induced cells perifused with the indicated solutions (fluorescence/minimum fluorescence [*F*/*F*_min_]). (**c**, **d**) Change in Fura2-AM fluorescence at 2.5 mmol/l extracellular Ca^2+^ (Ca^2+^_EC_) (**c**) and 0 mmol/l Ca^2+^_EC_ (**d**) in induced cells relative to not-induced cells. (**e**) Analysis of the time to Ca^2+^_C_ peak post-CPA addition. (**f**, **g**) Analysis of CPA-mediated Ca^2+^_ER_ leak upslope (**f**) and downslope after peak (**g**). (**h**) Ca^2+^_ER_ leak AUC post-CPA addition for TALK-2-expressing cells vs not-induced cells. (**i**) Representative Ca^2+^_ER_ traces using GCEPIA-SNAP_ER_ for cells imaged in 0 mmol/l Ca^2+^_EC_ and thapsigargin-mediated Ca^2+^_ER_ store depletion (GCEPIA1 fluorescence/SNAP_ER_ fluorescence [*F*_GCEPIA1_/*F*_SNAP_]). (**j**) Fold change in Ca^2+^_ER_ from the TALK-2-expressing cells relative to not-induced cells at 0 mmol/l Ca^2+^_EC_. (**k**) Per cent fold change in the amplitude of the thapsigargin response in the TALK-2-induced cells relative to not-induced cells. (**l**) Analysis of the downslope of Ca^2+^_ER_ release upon addition of thapsigargin. (**m**) Representative Ca^2+^_C_ traces in response to removing Ca^2+^_EC_ in TALK-2-expressing and not-induced cells pretreated with either DMSO or CM-4620 (10 μmol/l). (**n**) Fold change in Ca^2+^_C_ with removal of Ca^2+^_EC_. (**o**) Representative ASAP3_ER_ traces for cells at 0 mmol/l Ca^2+^_EC_ and addition of thapsigargin (fluorescence/fluorescence at 200 s [*F*/*F*_200_]). (**p**) The amplitude of ER *V*_m_ hyperpolarisation in response to thapsigargin. *n*=3 biological replicates for all experiments; *p<0.05, **p<0.01,***p<0.001 (by one-sample *t* test [**c**, **d**, **j**, **k**, **p**], one-way ANOVA [**n**] or unpaired Student’s *t* test [**e**–**h**, **l**]). a.u., arbitrary units; I, induced; NI, not induced; Tg, thapsigargin

Because ER-localised K^+^ channels enhance Ca^2+^_ER_ release [18, 25, 44], we next examined whether TALK-2 modulates Ca^2+^_ER_. This was accomplished using a variety of indicators for Ca^2+^_C_ (Fura-2 AM), Ca^2+^_ER_ (GCEPIA1-SNAP_ER_) and ER *V*_m_ (ASAP3_ER_) (Fig. 5a) Stimulation of Ca^2+^_ER_ leak with sarco/endoplasmic reticulum Ca^2+^ ATPase (SERCA) inhibition by cyclopiazonic acid (CPA) resulted in threefold less Ca^2+^_C_ elevation in cells expressing TALK-2 vs control cells (*n*=3; Fig. 5b, h). TALK-2 expressing cells showed increased Ca^2+^_C_ relative to not-induced cells, likely due to enhanced Ca^2+^_ER_ leak under basal conditions (0.12 ± 0.02-fold Ca^2+^_C_ increase in TALK-2-expressing cells vs control cells; *n*=3; Fig. 5c and ESM Fig. 1). However, this was not observed with removal of extracellular Ca^2+^ (0.009 ± 0.016 fold increase in TALK-2 expressing cells vs control cells; *n*=3; Fig. 5d). To determine whether TALK-2 reduced Ca^2+^_ER_ storage, we used an ER-localised Ca^2+^ indicator (GCEPIA1-SNAP_ER_), which allows normalisation of GCEPIA1 to expression (monitored by SNAP-Cell 647-SiR) [41]. Indeed, TALK-2-expressing cells showed less Ca^2+^_ER_ storage (16.7 ± 0.5% reduction in Ca^2+^_ER_ in TALK-2-expressing cells; Fig. 5j). TALK-2 also blunted Ca^2+^_ER_ leak following SERCA inhibition with CPA (ΔCa^2+^_ER_ amplitude: 24.3 ± 3.8% reduced in TALK-2-expressing cells; *n*=4; ESM Fig. 2) or thapsigargin (ΔCa^2+^_ER_ amplitude: 25 ± 5% reduced in TALK-2-expressing cells; *n*=3; Fig. 5k). The changes in Ca^2+^_ER_ following Ca^2+^_ER_ leak did not result in a change in luminal pH when thapsigargin was used to inhibit SERCA (ESM Fig. 3) [45]. This suggests that changes in TALK-2 activity or GCEPIA1 fluorescence were independent of ER luminal pH. Furthermore, CPA-induced time to Ca^2+^_C_ peak was reduced (induced cells 58.67 ± 3.85 s vs control cells 112.3 ± 14.3 s; Fig. 5e) and the resulting slope was increased in cells expressing TALK-2 vs control cells (induced 14.81 ± 0.75 vs control 8.90 ± 1.30; *n*=4; Fig. 5e–g). This suggests that TALK-2 enhances the rate of Ca^2+^_ER_ leak, which could be due to dissipation of negative charge that builds up on the intraluminal side of the ER membrane during Ca^2+^_ER_ release. The observation that TALK-2-expressing cells show a drop in Ca^2+^_C_ when switching from 2.5 mmol/l Ca^2+^ to 0 mmol/l Ca^2+^ suggested that store-operated Ca^2+^ entry (SOCE) may be elevated due to depletion of Ca^2+^_ER_ stores. To address this, Orai1 and Orai2 channels were inhibited with CM-4620, which diminished SOCE elevation following Ca^2+^_ER_ depletion (ESM Fig. 4). CM-4620 inhibition of SOCE in TALK-2-expressing cells prevented the drop in Ca^2+^_C_ following a switch from 2.5 mmol/l Ca^2+^ to 0 mmol/l Ca^2+^; therefore, at 2.5 mmol/l Ca^2+^ TALK-2 expressing cells treated with CM-4620 (I-CM-4620) decreased Ca^2+^_C_ by 4.6 ± 0.4% compared with I-DMSO (Fig. 5m, n). This suggests that SOCE is elevated by TALK-2-mediated reduction of Ca^2+^_ER_ storage.

We next examined whether TALK-2 regulates Ca^2+^_ER_ leak-mediated ER membrane hyperpolarisation with ASAP3_ER_ [39]. To confirm that a reduction in ASAP3_ER_ correlates with ER membrane hyperpolarisation, TALK-2 expressing and control T-REx-293 cells expressing ASAP3_ER_ were permeabilised with digitonin in solution with 0 mmol/l Ca^2+^ and 4.7 mmol/l K^+^, which caused a rapid drop in fluorescence (ESM Fig. 5). This hyperpolarisation was caused by both Ca^2+^_ER_ release and K^+^ efflux from the ER. TALK-2 expression reduced the amplitude of Ca^2+^_ER_ leak-mediated ER *V*_m_ hyperpolarisation (ΔASAP3_ER_ amplitude following thapsigargin treatment: decreased by 20.0 ± 0.8% in TALK-2-expressing cells relative to not-induced cells); *n*=4; Fig. 5o, p). This supports the notion that the electrical driving force for Ca^2+^_ER_ leak is enhanced by TALK-2-dependent limitation of ER *V*_m_ hyperpolarisation.

**Fig. 6:**
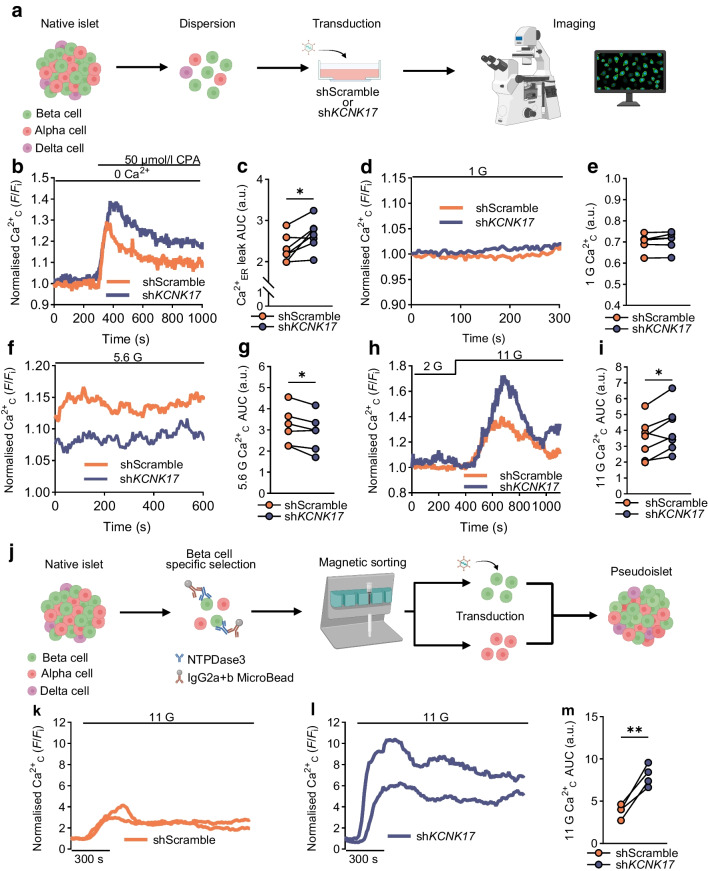
TALK-2 reduces Ca^2+^_ER_ storage and elevates Ca^2+^_C_ at basal glucose but limits Ca^2+^_C_ at high glucose. (**a**) Schematic depicting human islet dispersion, transduction and plating for Ca^2+^ imaging (created in BioRender. Dobson, J. (2026) https://BioRender.com/5bqhh65). (**b**) Representative Ca^2+^_C_ traces of human beta cells transduced with either shScramble or sh*KCNK17* imaged at 0 mmol/l extracellular Ca^2+^ (Ca^2+^_EC_) baseline and Ca^2+^_ER_ leak into the cytoplasm mediated by CPA (fluorescence/initial fluorescence [*F*/*F*_i_]). (**c**) Analysis of the AUC for CPA-mediated Ca^2+^_ER_ leak. (**d**, **f**, **h**) Representative Ca^2+^_C_ traces of human beta cells transduced with either shScramble or sh*KCNK17* and imaged at 1 mmol/l glucose (**d**) and 5.6 mmol/l glucose (**f**), and from 2 mmol/l to 11 mmol/l glucose (**h**). (**e**) Ca^2+^_C_ analysis of shScramble vs sh*KCNK17* transduced beta cells imaged at 1 mmol/l glucose. (**g**, **i**) Ca^2+^_C_ AUC in response to 5.6 mmol/l glucose (**g**) or 11 mmol/l glucose (**i**) in shScramble vs sh*KCNK17* transduced beta cells. (**j**) Schematic depicting human pseudoislet generation with beta cell specific knockdown of TALK-2 (created in BioRender. Dobson, J. (2026) https://BioRender.com/djkhtzb). (**k**) Representative Ca^2+^_C_ traces (GCaMP6s) from shScramble pseudoislets stimulated with 11 mmol/l glucose. (**l**) Representative Ca^2+^_C_ traces (GCaMP6s) from sh*KCNK17* pseudoislets stimulated with 11 mmol/l glucose. (**m**) AUC analysis for the 11 mmol/l response from shScramble vs sh*KCNK17* pseudoislets. Each donor is represented by two dots connected by a line: one dot for shScramble beta cells and the other for sh*KCNK17* beta cells. *n*=4 (**m**), 5 (**e**, **g**) or 7 (**c**, **i**) donors.*p<0.05, **p<0.01 (by paired *t* test [donor matched; **c**, **e**, **g**, **i**, **m**]). a.u., arbitrary units; 1 G, 1 mmol/l glucose; 2 G, 2 mmol/l glucose; 5.6 G, 5.6 mmol/l glucose; 11 G, 11 mmol/l glucose

The effect of TALK-2 on human beta cell Ca^2+^_ER_ was determined in dispersed islet insulin-positive cells with (sh*KCNK17*) or without (shScramble) TALK-2-KD. TALK-2 was found to limit Ca^2+^_ER_ leak following SERCA inhibition, suggesting that human beta cell TALK-2 reduces Ca^2+^_ER_ storage by promoting Ca^2+^_ER_ leak (CPA AUC: shScramble 2.30 ± 0.12 vs sh*KCNK17* 2.65 ± 0.14; *n*=7; Fig. 6a–d and ESM Fig. 6). Muscarinic signalling was assessed to determine whether TALK-2 contributes to Ca^2+^_ER_ release through inositol trisphosphate receptors (IP3Rs). Interestingly, pseudoislets with or without beta cell-specific TALK-2-KD showed equivalent acetylcholine-stimulated Ca^2+^_C_ elevation (shScramble 93.58 ± 14.01 vs sh*KCNK17* 75.95 ± 7.52; *n*=4 donors; ESM Fig. 7a–c). Ca^2+^_ER_ release was also measured via activation of ryanodine receptors (RyRs) with 10 mmol/l caffeine. INS-1(823/13) cells were transduced with either wild-type (WT) *KCNK17* or dominant negative *KCNK17* (RIP adenovirus containing a mKate2 reporter), and displayed similar RyR-mediated Ca^2+^_C_ increases (ESM Fig. 7d–f). Taken together, this suggests that TALK-2 promotes Ca^2+^_ER_ leak but not IP3R or RyR Ca^2+^_ER_ release, possibly due to the biophysical properties of Ca^2+^_ER_ release channels or the proximity of TALK-2 to Ca^2+^_ER_ leak channels [39].

### TALK-2 elevates beta cell basal Ca^2+^_C_ levels and reduces glucose-stimulated Ca^2+^_C_ entry

We next evaluated whether TALK-2 channels modulate human beta cell Ca^2+^_C_ handling. TALK-2-KD caused an increase in dispersed beta cell Ca^2+^_C_ at 11 mmol/l glucose (shScramble 3.45 ± 0.48 vs sh*KCNK17* 4.07 ± 0.55; *n*=7; Fig. 6h, i and ESM Fig. 8c), a reduction in Ca^2+^_C_ at 5.6 mmol/l glucose (shScramble 3.16 ± 0.36 vs sh*KCNK17* 2.88 ± 0.36; *n*=6; Fig. 6f, g and ESM Fig. 8b) and no difference in Ca^2+^_C_ at 1 mmol/l glucose (shScramble 0.699 ± 0.017 vs sh*KCNK17* 0.706 ± 0.018; *n*=6; Fig. 6d, e and ESM Fig. 8a). Glucose-stimulated beta cell Ca^2+^_C_ entry was also greater in the TALK-2-KD pseudoislets (11 mmol/l glucose; shScramble 3.84 ± 0.40 vs sh*KCNK17* 8.01 ± 0.64; *n*=4; Fig. 6k, m). This suggests that TALK-2 enhancement of Ca^2+^_ER_ leak elevates basal Ca^2+^_C_. However, as TALK-2 reduced Ca^2+^_ER_ stores, this enhances Ca^2+^_ER_ storage capacity. Thus, TALK-2-mediated increase in Ca^2+^_ER_ storage capacity enhances Ca^2+^_ER_ uptake during SERCA activation, which limits glucose-stimulated Ca^2+^_C_.

### TALK-2 promotes basal insulin secretion and limits glucose-stimulated insulin secretion

**Fig. 7:**
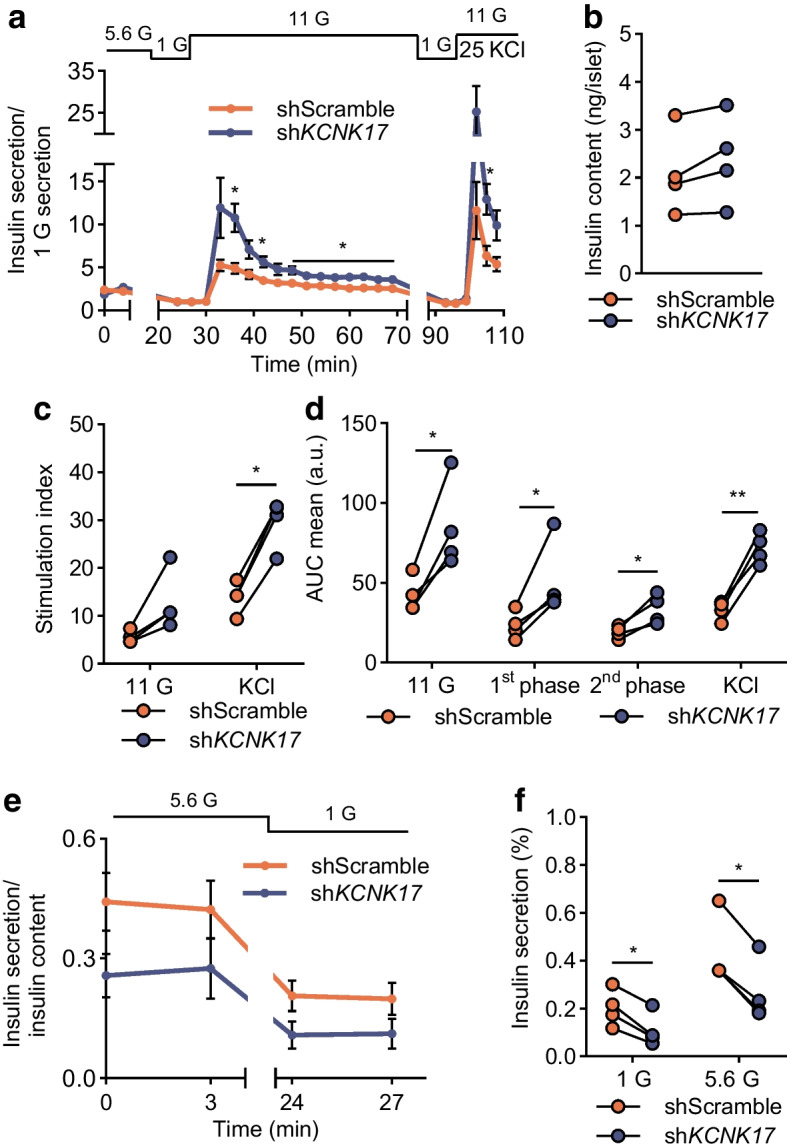
TALK-2 reduced insulin secretion at high glucose and increased insulin secretion at low glucose. (**a**) Dynamic insulin secretion under the indicated conditions from human pseudoislets containing either shScramble or sh*KCNK17* transduced beta cells. Traces represent a mean of four human donors normalised to insulin secretion taken at 27 min at 1 mmol/l glucose. (**b**) Comparison of the insulin content per islet between shScramble vs sh*KCNK17* pseudoislets. (**c**) Stimulation index at 11 mmol/l glucose and 25 mmol/l KCl determined by dividing the peak insulin secretion by the initial insulin secretion of the response. (**d**) AUC from parts of (**a**). First-phase insulin secretion was considered as the first 10 min post-11 mmol/l glucose with second-phase insulin secretion being the remainder of time in 11 mmol/l glucose. (**e**) Dynamic insulin secretion at 5.6 mmol/l glucose and 1 mmol/l glucose when normalised to insulin content. (**f**) Analysis of (**e**) by taking the mean insulin secretion at 5.6 mmol/l and 1 mmol/l glucose. *p<0.05, **p<0.01 (by paired *t* test). *n*=4 donors. 1 G, 1 mmol/l glucose; 5.6 G, 5.6 mmol/l glucose; 11 G, 11 mmol/l glucose; 25 KCl, 25 mmol/l KCl

As Ca^2+^ influx is required for insulin secretion, we next examined how TALK-2 modulates insulin secretion from pseudoislets with or without beta cell-selective TALK-2-KD. TALK-2-KD significantly increased pseudoislet glucose-stimulated insulin secretion (GSIS) at 11 mmol/l glucose (shScramble 42.53 ± 5.52 sh*KCNK17* 85.01 ± 13.96; *n*=4; Fig. 7a, d and ESM Fig. 9b). However, TALK-2-KD reduced pseudoislet insulin secretion at 1 mmol/l (shScramble 0.2024 ± 0.0389 vs sh*KCNK17* 0.1096 ± 0.0356; *n*=4; Fig. 7e, f and ESM Fig. 9d) and 5.6 mmol/l glucose (shScramble 0.432 ± 0.073 vs sh*KCNK17* 0.266 ± 0.065; *n*=4; Fig. 7e, f and ESM Fig. 9c). Furthermore, there was a ~twofold increase in the insulin stimulation index and AUC for the KCl-mediated depolarisation response of TALK-2-KD pseudoislets (Fig. 7c, d and ESM Fig. 9a). The data suggest that TALK-2-mediated elevation of basal Ca^2+^_C_ increases insulin secretion and that reduction of glucose-stimulated Ca^2+^_C_ limits GSIS. Importantly, the data suggest that greater TALK-2 activity would increase basal insulin secretion and reduce GSIS; this could contribute to greater risk for developing type 2 diabetes due to associated SNPs in or near *KCNK17*.

## Discussion

Polymorphisms in or around the gene encoding TALK-2 indicate that elevated TALK-2 activity and/or expression in human islets increase the risk of type 2 diabetes. Here we found that TALK-2 localises and forms functional channels on the ER membrane. These currents depolarised the ER membrane during and following Ca^2+^_ER_ release, which enhanced Ca^2+^_ER_ leak. Importantly, as TALK-2 elevated basal insulin secretion and limited GSIS, individuals with *KCNK17* SNPs may have greater insulin resistance and reduced GSIS. Together, these data suggest that TALK-2 inhibitors could be used to elevate beta cell Ca^2+^_ER_, limit basal insulin secretion and promote GSIS in individuals with type 2 diabetes.

Ca^2+^_ER_ is a critical regulator of insulin production, secretion and response to diabetogenic stress [1, 4, 46, 47]. Due to the importance of beta cell Ca^2+^_ER_, specific ion channels serve to provide precise orchestration of Ca^2+^_ER_ storage and release, including a subset of ER-localised K2P channels [18]. Interestingly, fluorescently tagged TALK-2 K^+^ channels have been shown to resemble ER localisation [48]. Moreover, endogenous TALK-2 in a human beta cell line showed almost exclusive intracellular localisation [17]. Similarly, we observed clear intracellular TALK-2 localisation in human pancreatic beta cells and in cells overexpressing TALK-2. Considering that all ion channels move through the ER membrane and many do not show function there (e.g. K_ATP_) [49], K2P channel activity on the ER membrane could be due in part to ER localisation or structural determinants of these channels [18]. Indeed, TALK-2 shows prominent ER localisation and significant intracellular staining in human beta cells. Interestingly, TALK-1 and TALK-2 currents recorded on the ER membrane do not show the typical outward rectification seen on the plasma membrane. This is likely due to electrophysiological recording conditions that alter the ion occupancy of the ER luminal pore of these channels. The pipette and recording solutions contained high K^+^, which has been shown to eliminate plasma membrane TALK-2 outward rectification [16]. These recording solutions also eliminated any K^+^ gradient across the ER membrane; this was done because the mechanism for how and whether an ER K^+^ concentration gradient is generated has not been established. It is important to note that even without a K^+^ concentration gradient across the ER membrane, Ca^2+^_ER_ release will still lead to an electrical driving force for luminal influx of K^+^ through TALK-2 channels. The intracellular staining pattern and ER localisation of TALK-2 closely resembles that of TALK-1 [14]. TALK-1 and TALK-2 can form functional channels as either monomers or heterodimers and thus their similar patterns of localisation may be attributable to heterodimerisation [17]. While heterodimeric TALK-1/TALK-2 channels have been recorded at the plasma membrane, the biophysical activity of these heterodimers on the ER membrane remains to be determined [17]. A potential caveat with previous approaches using TALK-1 dominant negative constructs is that the changes in beta cell Ca^2+^_ER_ storage could be due in part to inhibition of both TALK-1 and TALK-2 channels [18, 50]. However, the selective beta cell *KCNK17* shRNA approach used here indicates an important role for ER-localised TALK-2 either as homodimers or heterodimers. Taken together, this suggests that TALK-1 and TALK-2 serve functional roles on the ER that would be predicted to set beta cell Ca^2+^_ER_ storage and promote Ca^2+^_ER_ release.

Beta cell Ca^2+^_ER_ depletion results in stromal interaction molecule 1 (STIM1) translocation to the plasma membrane and subsequent activation of SOCE through Orai channels, which is likely altered by TALK-2-mediated depletion of Ca^2+^_ER_ stores. Indeed, SOCE was elevated in TALK-2-expressing cells leading to greater basal Ca^2+^_C_ that was lost with removal of extracellular Ca^2+^. This suggests that TALK-2 enhancement of Ca^2+^_ER_ leak elevates basal Ca^2+^_C_ in part by increasing SOCE. Interestingly, STIM1 translocation to the plasma membrane not only initiates SOCE but also interacts with L-type Ca^2+^ channels leading to suppression of the L-type current [51]. Thus, TALK-2-mediated reduction of Ca^2+^_ER_ stores could potentially reduce L-type Ca^2+^ channel activity leading to the observed increase of glucose-stimulated Ca^2+^ influx in TALK-2-KD beta cells. It is also important to note that depletion of Ca^2+^_ER_ stores following beta cell SERCA2 ablation results in diminished glucose-stimulated Ca^2+^_C_ influx, further supporting this concept [21]. TALK-2 depletion may also elevate human beta cell Ca^2+^_ER_ to a level that limits further Ca^2+^_ER_ uptake during glucose-stimulated Ca^2+^_C_ influx. This would be predicted to amplify glucose-stimulated Ca^2+^_C_ influx; similar to amplification of glucose-stimulated Ca^2+^_C_ influx following acute SERCA inhibition of Ca^2+^_ER_ uptake [52]. While this suggests an important role for TALK-2 control of Ca^2+^_C_ in modulating glucose-stimulated Ca^2+^_C_ influx, the elevation in Ca^2+^_C_ in response to SERCA inhibition is only modestly increased with TALK-2-KD. This could be due to the loss of TALK-2-mediated countercurrent to enhance Ca^2+^ leak, thus not allowing complete depletion of Ca^2+^_ER_ stores. Indeed, Ca^2+^_ER_ levels are always elevated even during SERCA inhibition in cells without TALK-2 expression compared with cells with TALK-2 expression. Interestingly, SERCA-mediated elevations of human beta cell Ca^2+^ result in a very slow return to baseline. Therefore, human beta cells show either prolonged Ca^2+^_ER_ leak or slower Ca^2+^_C_ extrusion following Ca^2+^_ER_ leak [18]. A potential mechanism for this could be the Ca^2+^_ER_ buffering capabilities of beta cell ER by chaperone proteins, which may slowly release bound Ca^2+^_ER_ over time and contribute to the modest increases in Ca^2+^_C_ in response to SERCA inhibition with TALK-2-KD. Another possibility is due to the slow rate of plasma membrane Ca^2+^ extrusion proteins activated with Ca^2+^_ER_ leak (such as plasma membrane Ca^2+^ ATPase). The amount of countercurrent activated during Ca^2+^_ER_ leak could also be greater in cells with rapid return to baseline.

ER-localised K^+^ channels have been predicted to increase the driving force for Ca^2+^_ER_ release by moving the ER membrane potential away from the equilibrium potential of Ca^2+^ channels [25, 26, 44]. Charge buildup occurs on membranes due to their thin makeup and ion impermeability. Thus, when Ca^2+^_ER_ channels open, Ca^2+^ efflux is balanced by negative charge accumulation on the luminal membrane. Indeed, ER *V*_m_ reporters show that Ca^2+^_ER_ release via either RyR activation or SERCA inhibition with thapsigargin hyperpolarises the ER membrane [39]. Similarly, we observed ER *V*_m_ hyperpolarisation following SERCA inhibition, limited in cells expressing TALK-2. Furthermore, we found that ER TALK-2 channels were more active at hyperpolarised voltages. Thus, hyperpolarisation following Ca^2+^_ER_ release may be required to activate TALK-2 channels. Other K^+^ channels, such as BK channels, have been shown to limit the spread of ER hyperpolarisation following Ca^2+^_ER_ release [39]. However, BK channels have a larger conductance and are not constitutively active, thus K2P channels would be predicted to serve as a setpoint for ER *V*_m_ based on their voltage dependence of activation [53, 54]. Interestingly, we observed no TALK-2-induced changes in IP3R or RyR-mediated Ca^2+^_ER_ release, possibly attributable to a potentially closer location of TALK-2 to Ca^2+^_ER_ leak channels. Indeed, RyRs interact with and are controlled by TRIC-A and TRIC-B K^+^ channel countercurrents [27, 55]; this suggests that specific K^+^ channels localise with and provide countercurrents for certain Ca^2+^_ER_ release and leak channels. RyRs are permeable not only to Ca^2+^ but also to other cations such as K^+^, which has been proposed to provide a countercurrent that helps maintain the driving force for RyR-mediated Ca^2+^_ER_ release [56–58]. Furthermore, there is no impact of IP3R activation on ER *V*_m_ [39]. A lack of ER *V*_m_ change following IP3R activation could be due to a receptor-dependent mechanism or subunit complex (e.g. countercurrent) that prevents ER *V*_m_ hyperpolarisation. Future studies will examine how the specific biophysical characteristics of K2P channels control ER *V*_m_ and the specific Ca^2+^_ER_ release mechanisms that they regulate.

Although this is the first demonstration of TALK-2 channels on the ER membrane, TALK-2 channels are also expressed and are functional on the plasma membrane [8–10, 14, 17, 48]. The TALK subfamily of K2P channels are outwardly rectifying leak K^+^ channels classified by their extracellular alkaline sensitivity [14, 16]. Of the three TALK channels, TALK-2 requires supraphysiological alkaline conditions for activation. Similarly, we found a significant increase in TALK-2 activity at alkaline pH (8.8). The exact mechanism(s) that promote alkaline pH conditions required for plasma membrane TALK-2 activity remain(s) to be determined. One such mechanism initiating alkaline activation of TASK-2 channels is their proximity to bicarbonate transporters, which provide a local alkaline microenvironment near the channel [59]. *KCNK17* is also expressed in the intestinal tract, and this location can reach alkaline pHs that may activate plasma membrane TALK-2. Interestingly, plasma membrane K2P currents from beta cells with TALK-2-KD did not show a difference compared with controls at either physiological or alkaline pH. This suggests that either TALK-2 channels are not functional at the beta cell plasma membrane or that the loss of TALK-1/TALK-2 heterodimers leads to TALK-1 homodimer currents that compensate for the loss of TALK-2 subunits. In cardiac cells, plasma membrane TALK-2 currents have been recorded and GOF mutation or polymorphism leads to alterations in electrical activity, protecting against long QT syndrome 2 severity or promoting severe cardiac conduction disorder [8, 48]. An important question arising from this work is why beta cell TALK-2 channels are active on the ER membrane vs the plasma membrane. Membrane architecture may be responsible (e.g. the thinner nature and greater fluidity of the ER membrane may reduce TALK-2 alkaline sensitivity). Importantly, TALK-2 likely serves roles in cardiac sarcoplasmic reticulum Ca^2+^ release and storage. Therefore, future studies are required to determine the conditions that activate beta cell plasma membrane TALK-2 and whether TALK-2 GOF disrupts cardiac contraction by promoting sarcoplasmic reticulum Ca^2+^ release and depletion.

*KCNK17* SNPs associated with an increased risk of developing type 2 diabetes elevate expression and/or function of TALK-2 [7]. Interestingly, the rs1535500 SNP that results in TALK-1 GOF is in high linkage disequilibrium with four SNPs in the promoter and coding sequence of *KCNK17* [7, 15]. Thus, beta cell dysfunction during the pathogenesis of type 2 diabetes may be due not only to overactive TALK-1 but also to increased TALK-2 expression and activity. Moreover, one of these SNPs in *KCNK17* disrupts regulatory factor F6 (RFX6) binding, which may result in islet specific overexpression of TALK-2 [7, 60]. Based on TALK-2 depletion of Ca^2+^_ER_ storage, it is interesting to speculate that increased TALK-2 expression or activity would promote ER stress under conditions of diabetogenic stress. Another potential role for the association of TALK-2 with type 2 diabetes could arise from its alteration of insulin secretion. Indeed, we find that TALK-2 plays an important role limiting GSIS but promoting basal insulin secretion. Thus, polymorphisms that increase TALK-2 would be predicted to elevate basal insulin secretion under diabetogenic conditions and potentially promote insulin resistance. However, *KCNK17* SNPs have not been associated with fasting insulin in GWAS. Furthermore, TALK-2-mediated reduction of GSIS would be predicted to elevate postprandial glucose levels during the pathogenesis of type 2 diabetes. Individuals carrying *KCNK17* risk SNPs might respond favourably to bedtime diazoxide treatment, which induces night-time beta cell rest and lowers basal insulin secretion [61, 62].

While this study provides clear evidence that TALK-2 regulates beta cell Ca^2+^ handling and insulin secretion, there are several limitations. First, although we were unable to record TALK-2 channels at the plasma membrane, they may still serve active roles there. Indeed, overexpression of TALK-2 allows functional currents at the plasma membrane [8–10, 14, 16, 48]. Thus, future studies are required to identify conditions that permit physiological activation of plasma membrane TALK-2 channels. Second, our data clearly shows that TALK-2 regulates Ca^2+^_ER_ release from human beta cells. However, the data only examined TALK-2 channel-mediated ER *V*_m_ depolarisation in HEK293 cells. Therefore, future studies are required to confirm that TALK-2 promotes human beta cell Ca^2+^_ER_ release by depolarising the ER *V*_m_. Furthermore, although we found that TALK-2 modulated ER voltage sensor fluorescence, these sensors are sensitive to pH and as a single-wavelength indicator they are sensitive to expression artefact. To overcome these obstacles, we used conditions that did not change ER pH and equivalent transfection conditions to enable similar expression. However, future studies with ratiometric ER voltage indicators will provide more detailed analysis of TALK-2 control of ER *V*_m_. Third, although glucose-stimulated Ca^2+^ entry was significantly increased in TALK-2-KD pseudoislets, this was accomplished using a single-wavelength genetically encoded Ca^2+^ indicator, GCaMP6s, which is prone to expression artefact. While the Ca^2+^ data recapitulated the observations in dispersed human beta cells imaged with the ratiometric probe Fura 2-AM, it will be important for future studies to evaluate TALK-2 Ca^2+^ handling in pseudoislets using ratiometric Ca^2+^ sensors. Fourth, this study used *KCNK17* shRNAs to specifically evaluate how TALK-2 regulates beta cell function. One limitation to this approach is that it does not allow examination of TALK-1/TALK-2 heterodimers that may control beta cell Ca^2+^ handling and insulin secretion. It is important for future studies to examine how TALK-1/TALK-2 heterodimers impact beta cell function and if TALK-2 GOF or increased expression alter heterodimer activity or abundance. Finally, both male and female donors were combined in the data analysis within this manuscript. Although we observed equivalent TALK-2 function in beta cells from either sex, a larger sample size is required to determine if there are any beta cell TALK-2 sex-dependent differences.

## Supplementary Information

Below is the link to the electronic supplementary material.

ESM (PDF 586 KB)

## Data Availability

The authors declare that all data supporting the findings of this study are available and will be shared upon request. The eQTL dataset used in this study was obtained from Alonso et al [32], and the corresponding summary statistics are available through the TIGER portal, https://tiger.bsc.es/downloads. The multi-ancestry GWAS dataset was derived from Suzuki et al [31], with summary statistics accessible from the DIAGRAM consortium website, https://www.diagram-consortium.org/downloads.html.

## Abbreviations

ASAP3_ER_: Accelerated sensor of action potentials, ER-specific
Ca^2+^_C_: Cytoplasmic Ca^2+^
Ca^2+^_ER_: Endoplasmic reticulum Ca^2+^
CPA: Cyclopiazonic acid
eQTL: Expression quantitative trait locus
ER: Endoplasmic reticulum
GOF: Gain-of-function
GRP94: Glucose-related protein 94
GSIS: Glucose-stimulated insulin secretion
GWAS: Genome-wide association studies
IIDP: Integrated Islet Distribution Program
IP3R: Inositol trisphosphate receptor
K2P: Two-pore domain K^+^
qPCR: Real-time quantitative PCR
ROS: Reactive oxygen species
RyR: Ryanodine receptor
SERCA: Sarco/endoplasmic reticulum Ca^2+^ ATPase
SOCE: Store-operated Ca^2+^ entry
TALK: TWIK1-related alkalinisation-activated K^+^ channel
TRIC: Trimeric intracellular cation
UPR: Unfolded protein response
