# Supplementary material for: The diabetes-associated K+ channel TALK-2 controls human beta cell endoplasmic reticulum Ca2+ handling, which promotes basal insulin release and limits glucose-stimulated insulin secretion
Source: Diabetologia. 2026 Feb 25;69(6):1581–99. doi: 10.1007/s00125-026-06683-9 (PMC13109171; doi:10.1007/s00125-026-06683-9)
Supplement: Supplementary file 1 — ESM (PDF 586 KB) [file 125_2026_6683_MOESM1_ESM.pdf]

Electronic Supplementary Material

Donor ID	Sex	Age	Race	BMI	Experiments
SAMN31697847	M	30	W	31.2	qPCR
SAMN32537360	M	51	W	24.8	Ca^{2+} imaging
SAMN32875450	F	60	W	25.2	Ca^{2+} imaging, qPCR
SAMN33683883	M	48	W	28.5	Ca^{2+} imaging, qPCR
SAMN33902447	F	32	W	19.1	Ca^{2+} imaging
SAMN34204018	M	47	W	27.5	Ca^{2+} imaging
SAMN34356133	M	20	H	38.5	Ca^{2+} imaging
SAMN35301006	M	52	W	31.6	Ca^{2+} imaging
SAMN35848421	M	22	B	31.8	Ca^{2+} imaging
SAMN36471214	M	40	W	24.2	Ca^{2+} imaging
SAMN36845032	M	34	H	33	Ca^{2+} imaging
SAMN36823227	F	41	W	38.4	Ca^{2+} imaging
SAMN37068473	M	51	W	30.4	Ca^{2+} imaging
SAMN37158106	M	40	W	32.3	Ca^{2+} imaging
SAMN37350251	F	43	W	29.9	Ca^{2+} imaging
SAMN37638596	M	34	NH	27.2	Ca^{2+} imaging
SAMN37973608	F	55	H	31.7	Insulin secretion
SAMN38518088	F	57	H	30.1	Insulin secretion
SAMN39447621	M	49	H	34.4	Ca^{2+} imaging, Insulin secretion
SAMN39639106	M	49	B	30.6	Ca^{2+} imaging
SAMN39912961	M	39	w	31	Ca^{2+} imaging
SAMN39980165	F	60	W	29.5	Ca^{2+} imaging
SAMN40100962	M	58	W	28.8	Insulin secretion
SAMN40376066	F	34	W	25.7	Ca^{2+} imaging
SAMN40619409	M	63	B	34.1	Ca^{2+} imaging
SAMN41218531	M	38	W	29	Electrophysiology
SAMN41475017	F	63	W	24.9	Electrophysiology
SAMN43777471	M	57	W	28.8	Electrophysiology
SAMN44562976	F	62	W	38.4	Electrophysiology
SAMN44779828	F	59	W	26.3	Electrophysiology
SAMN45232023	F	55	W	29.6	Electrophysiology

ESM Table 1. Human islet donor descriptions used in this study.

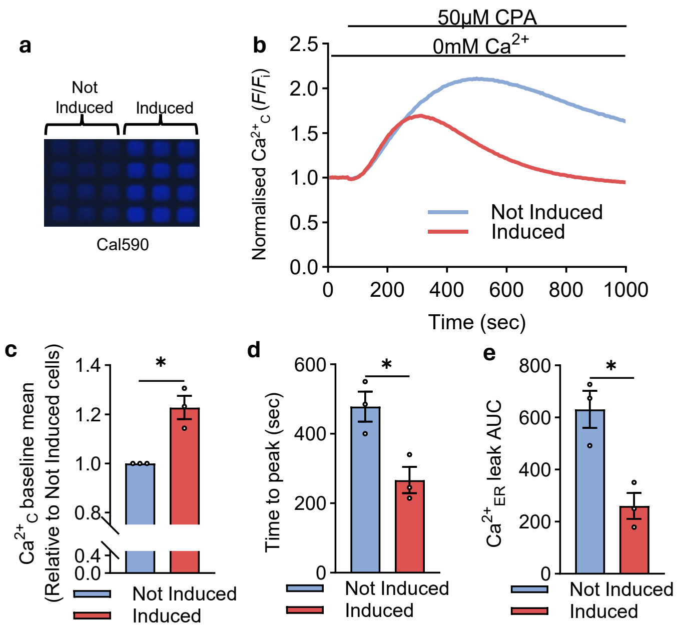

ESM Fig 1. TALK-2 expressing cells have diminished Ca^{2+}_{ER} stores and higher baseline Ca^{2+}_{C}. (**a**) Image of wells containing not induced and TALK-2 expressing cells (induced) loaded with Cal590 prior to Ca^{2+} imaging. (**b**) Representative Ca^{2+}_{EC} traces of CPA-mediated Ca^{2+}_{ER} release. (**c**) Analysis of relative baseline Ca^{2+}_{C} at 0mM Ca^{2+}_{EC} prior to CPA addition. (**d**) The time to CPA peak for not induced and TALK-2 expressing cells. (**e**) AUC of CPA-mediated Ca^{2+}_{ER} leak into the cytoplasm. *n*=3 biological replicates; Statistical analysis conducted using one sample T-test (**c**) or unpaired students T-test (**d-e**). *p<0.05

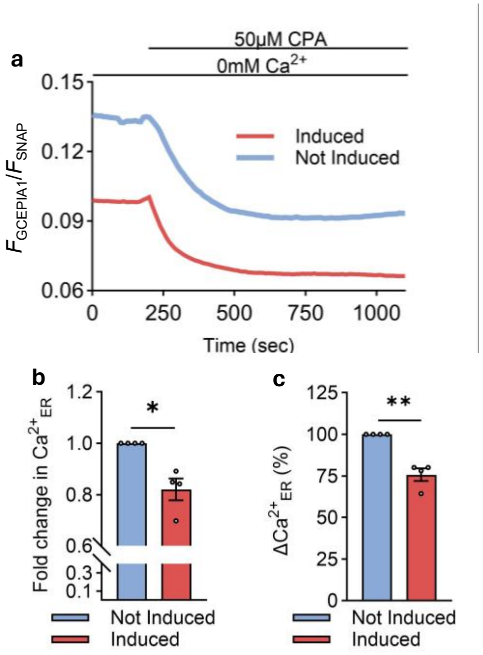

ESM Fig 2. TALK-2 expressing cells have depleted Ca^{2+}_{ER} stores. (**a**) Representative Ca^{2+}_{ER} traces in response to CPA-mediated Ca^{2+}_{ER} release. (**b**) Fold change analysis in Ca^{2+}_{ER} at 0mM Ca^{2+}_{EC} prior to CPA addition. (**c**) The percent change in Ca^{2+}_{ER} for not induced and TALK-2 expressing cells. *n*=4 biological replicates; statistical analysis conducted using one sample T-test. *p<0.05, **p<0.01

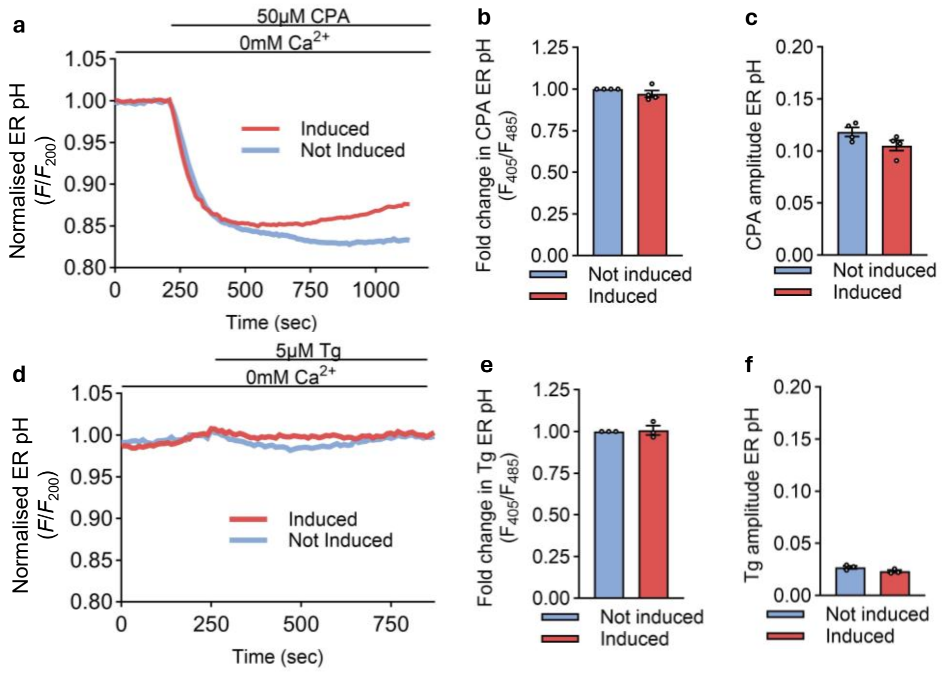

ESM Fig 3. ER pH reduces upon CPA addition but not Tg addition. (**a**) Representative ER pH changes in response to CPA from TALK-2 expressing and not induced cells. (**b**) Fold change in ER pH at 0mM Ca^{2+} relative to not induced cells. (**c**) Analysis of the ER pH amplitude in response to CPA. (**d**) Representative ER pH changes in response to thapsigargin (Tg) from TALK-2 expressing and not induced cells. (**e**) Fold change in ER pH at 0mM Ca^{2+} relative to not induced cells. (**f**) Analysis of the ER pH amplitude in response to Tg. *n*=4 and 3 biological replicates for CPA and Tg respectively; statistical analysis conducted using one sample T-test (b,e) or unpaired Student's T-test (**c,f**).

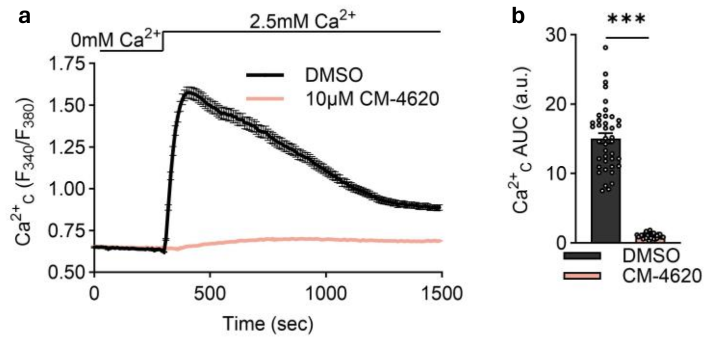

ESM Fig 4. CM-4620 diminished SOCE. (**a**) T-REx-293 cells preincubated with CPA and either CM-4620 or DMSO for 30 minutes and were then imaged in 0mM extracellular Ca^{2+} followed by the addition of 2.5mM Ca^{2+} to measure SOCE. (**b**) AUC analysis of the SOCE response in DMSO vs. CM-4620 treated cells. *n*= DMSO 40 cells, CM-4620 35 cells; statistical analysis conducted using Student's T-test. ***p<.001

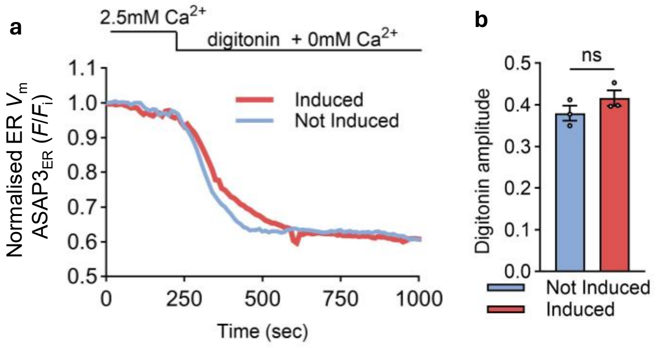

ESM Fig 5. ER V_m hyperpolarisation reduces $ASAP3_{ER}$ fluorescence. (**a**) Representative ER V_m traces from ER membrane hyperpolarisation with digitonin and 0mM Ca^{2+}. (**b**) Analysis of $ASAP3_{ER}$ amplitude in response to digitonin. *n*=3 biological replicates; statistical analysis conducted using unpaired Student's T-test.

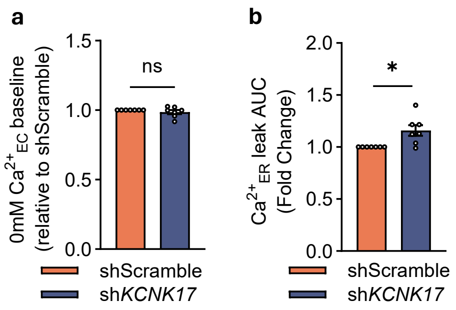

ESM Fig 6. Human beta cell TALK-2 limits Ca^{2+}_{ER} stores. (**a**) Ca^{2+}_{C} in sh*KCNK17* transduced beta cells at 0mM Ca^{2+}_{EC} relative to shScramble controls (one sample t-test from Fig. 6c). (**b**) Fold change in CPA-mediated Ca^{2+}_{ER} leak AUC relative to shScramble (one sample t-test from Fig. 6d). *n*= 7 donors; p<*0.05

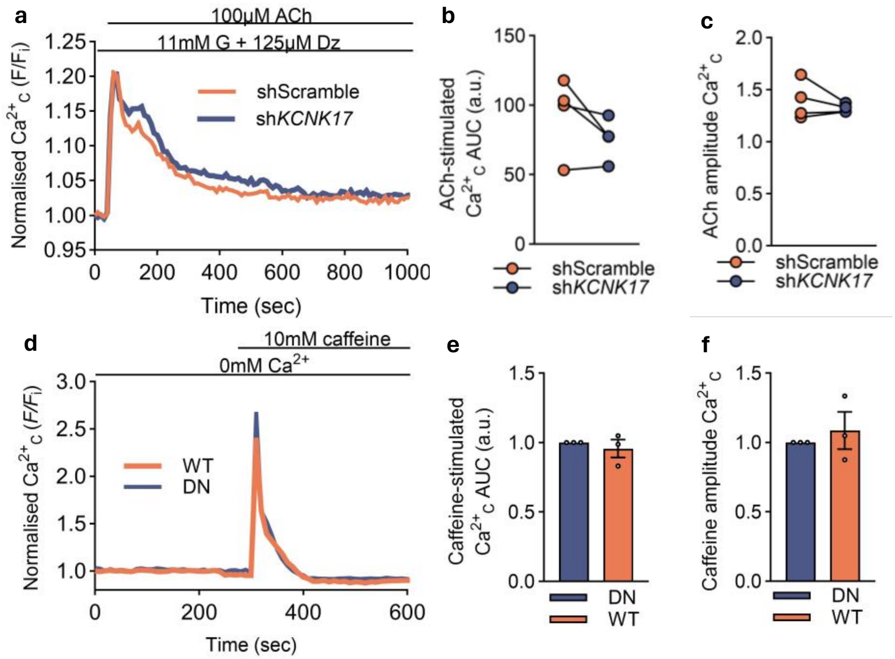

ESM Fig 7. TALK-2 expressing cells have diminished Ca^{2+}_{ER} stores and higher baseline Ca^{2+}_{C}. (**a**) Representative Ca^{2+} traces from human beta cells of IP3R-mediated Ca^{2+}_{ER} release. (**b**) AUC analysis of Ca^{2+}_{C} from IP3R-mediated Ca^{2+}_{ER} release stimulated by acetylcholine (Ach). (**c**) Analysis of Ca^{2+} amplitude in response to IP3R activation with Ach. (**d**) Representative Ca^{2+} traces from INS-1 (832/13) of RyR-mediated Ca^{2+}_{ER} release transduced with either dominant negative (DN) or wild-type (WT) TALK-2. (**e**) AUC analysis of Ca^{2+}_{C} from RyR-mediated Ca^{2+}_{ER} release stimulated by caffeine. (**f**) Analysis of Ca^{2+} amplitude in response to RyR activation with caffeine. *n*=4 donors (**b-c**) or 3 biological replicates (**e-f**); statistical analysis conducted using paired T-test (**b-c**) or one sample T-test (**e-f**).

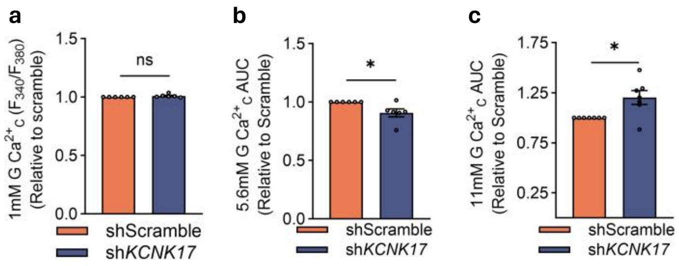

ESM Fig 8. TALK-2 promotes $Ca^{2+}{}_C$ entry at basal glucose but limits $Ca^{2+}{}_C$ entry at high glucose. Beta cell $Ca^{2+}{}_C$ at 1mM glucose (**a**), 5.6mM glucose (**b**), and 11mM glucose (**c**) relative to controls (one sample t-test from Fig. 6b, d, and f, respectively). *n*= 6 (**a,b**) or 7 donors (**c**); P<*0.05

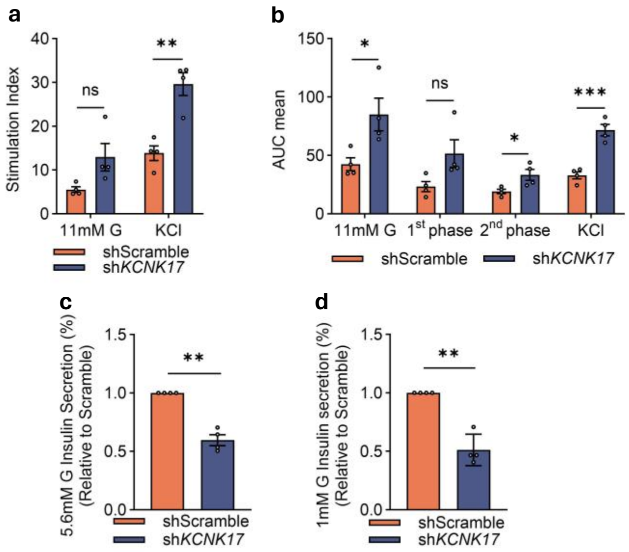

ESM Fig 9. TALK-2 limits insulin secretion under hyperglycemic conditions and promotes insulin secretion under euglycemic conditions. (**a**) Stimulation index of beta cell TALK-2-KD pseudoislets vs Scramble in response to 11mM glucose and 25mM KCl (multiple unpaired t-tests of fig. 8c.). (**b**) Mean insulin secretion AUC of pseudoislets under the specified conditions (1st phase = 1st ten minutes in 11mM glucose; 2nd phase = time after 10 minutes in 11mM glucose) (multiple unpaired t-tests of fig. 8d.). Insulin secretion of TALK-2-KD pseudoislets at 5.6mM glucose (**c**) and 1mM glucose (**d**) relative to Scramble controls (one sample t-test of fig. 8f.). *n*= 4 donors; P<*0.05
